# Transformation of waste cooking oil into C-18 fatty acids using a novel lipase produced by *Penicillium chrysogenum* through solid state fermentation

**DOI:** 10.1007/s13205-014-0268-z

**Published:** 2014-12-04

**Authors:** Sunil Kumar, Sangeeta Negi

**Affiliations:** Department of Biotechnology, Motilal Nehru National Institute of Technology, Teliyarganj, Allahabad, 221004 India

**Keywords:** Waste cooking oil, Chromatography, Lipase, Fatty acid

## Abstract

The prime aim of the current work was to illustrate the components existing in repeatedly used cooking oil and to develop an economical process for the production of fatty acids from low cost feedstock waste. The waste cooking oil was characterized by the occurrence of high molecular weight hydrocarbons and polymerized derivative of esters. Triacontanoic acid methyl ester, 2,3,5,8-Tetramethyldecane, 3,3 dimethyl heptane, and 2,2,3,3-teramethyl pentane were detected as thermal and oxidative contaminants that adversely affect the quality of cooking oil. Fundamentally, waste cooking oil comprises ester bonds of long chain fatty acids. The extracellular lipase produced from *P. chrysogenum* was explored for the hydrolysis of waste cooking oil. The incorporation of lipase to waste cooking oil in 1:1 proportion released 17 % oleic acid and 5 % stearic acid.

**Electronic supplementary material:**

The online version of this article (doi:10.1007/s13205-014-0268-z) contains supplementary material, which is available to authorized users.

## Introduction

Vegetable oil and ghee are chemically characterized as triglyceride molecules and widely used for the cooking purpose. (Maher and Bressler 2007). Repetitive use of cooking oil under elevated temperature causes deleterious changes in physical and chemical properties of oil by the process of hydrolysis, oxidation, and polymerization. The reactions occurring in repeated cooking of oil depend on original quality of oil, type of food materials, heating conditions and duration, and concentration of moisture and oxygen. High temperature and number of repeats decrease the flavor quality of oil and produce volatile or nonvolatile complexes. Most of volatile components evaporate and produce smoke that generates anoxygenic environment in working condition. The chemistry of nonvolatile compounds is complex and poorly understood process because of Diels–Alder and Amadori rearrangements (Martins et al. 2001). Therefore, it is not possible to predict the mechanism of formation of volatile or nonvolatile components due to isomerization, cyclization, thermal polymerization, hydrolysis and oxido-reduction process.

A huge amount of waste cooking oil generated from restaurant and food process industries is disposed without prior treatment. The Energy Information Administration in United States estimated that 100 million gallons of waste cooking oil is produced per day in USA (Radich 2006) while UK and EU countries generate approximately 200,000 tons and 700,000-1,000,000 tons of waste cooking in a year respectively. (Arjun et al. 2008). Theoretically, a single liter of oil can contaminate one million liters of water in spite of small volume ratio. These wastes have very high biological oxygen demand owing to highly reduced long chain fatty acids. Therefore, Environmental Protection Agency in 1994 released an ordinance under act of oil pollution in which animal fats and vegetable oils could not be exempted from regulations governing the cleanup of oil spills.

Rapidly advancing technologies and the use of custom-made biocatalysts have produced exciting opportunities for recycling of waste cooking oil. Transformation of waste cooking oil using enzymes makes process efficient in comparison to other physical and chemical treatments such as steam splitting and alkaline hydrolysis (Murty et al. 2002). Enzymes are recognized as highly selective and specific catalysts which accelerate the rate of reactions under mild conditions. Lipases (E.C. 3.1.1.3) are the members of the hydrolase family and exhibit positional specificity toward fatty acid and fatty acid selectivity in acylglycerol. Lipases hydrolyze the acylglycerol into fatty acids and glycerol. Lipases are unique biocatalysts that not only hydrolyze carboxylic ester bonds but also exploit to catalyze synthesis of fine chemicals through esterification, interesterification and transformations of racemic compounds. Lipase-mediated transformation of waste cooking oil could be a promising method for cost-effective production of FFA because 1.6 × 10^6^ tons of fatty acids are produced every year from various plant and animal sources through lipases (Pronk et al. 1988). The current study opened a new avenue to generate C-18 fatty acids from waste cooking oil by lipase for various industrial applications.

## Materials and methods

### Enzyme production for hydrolysis of waste cooking oil

A locally isolated strain of *P. chrysogenum* SNP5 was explored for the production of extracellular lipase using grease waste, wheat bran and Czepek-dox media (1:1:2 w/w) as substrate. Enzyme was extracted after 7 days of solid state fermentation (SSF) and centrifuged at 8,000 rpm for 15 min. The clear supernatant was used as a crude extracellular lipase enzyme (Kumar et al. 2011). A 1:1 ratio of emulsified cooking oil waste and lipase was mixed to mediate lipolytic reaction of cooking oil (Kumar et al. 2012).

### Gas chromatography

Fatty acids were methylated with BF_3_-MeOH as described by O’Fallon et al. (2007). The analytical conditions of GC for FAME detection were 75 m × 0.18 mm SP-2560 capillary column. Nitrogen gas was used as mobile phase at rate of 20 ml/min. The injector temperature was set at 250 °C and detector temperature at 260 °C using flame ionization detector (FID). Oven temperature was programmed from 140 to 240 °C at 4 °C/min.

## Results and discussion

### Characterization of waste cooking oil

**Fig. 1:**
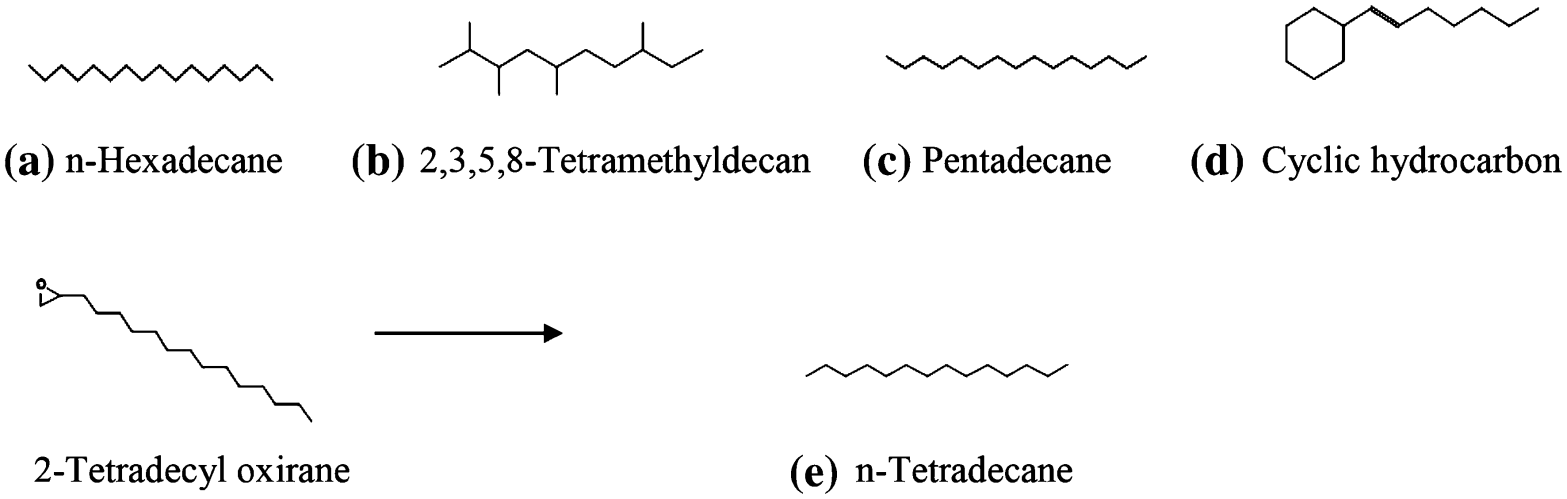
Cyclic and noncyclic derivative of hydrocarbon in waste cooking oil

**Fig. 2:**
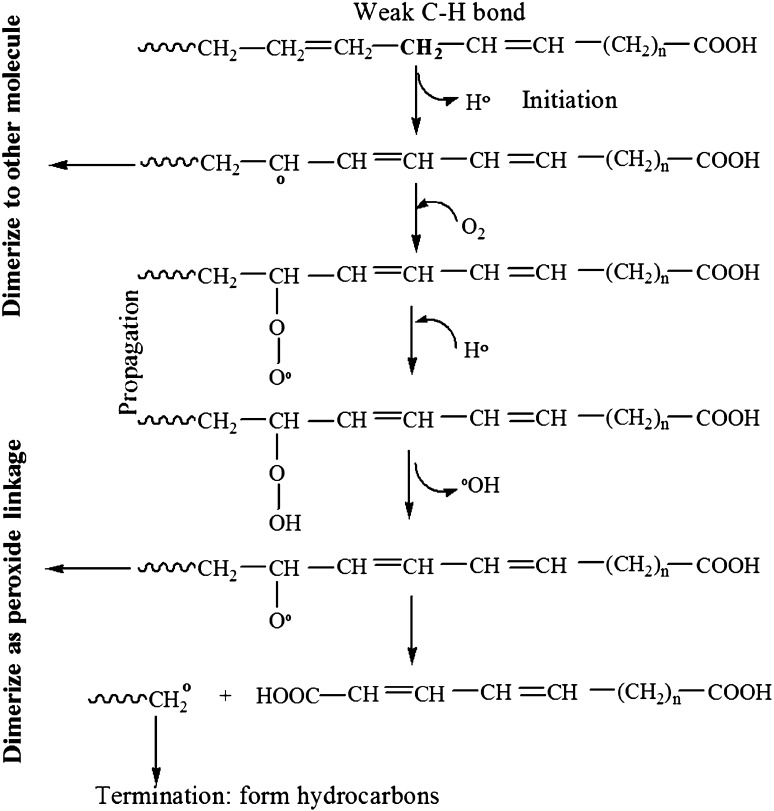
Proposed free radical mechanism: formation of hydrocarbon and polymerized derivation under process of thermal oxidation

**Fig. 3:**
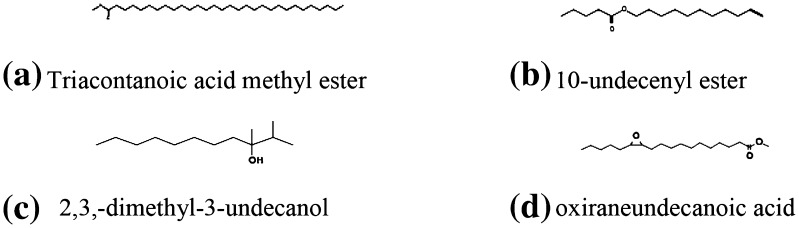
Oxidized and polymerized derivative of glycerides under elevated temperature

The thermal and oxidative changes of vegetable oil under cooking temperature were studied by GC–MS to depict the chemical constituent of waste cooking oil. GC–MS analysis of waste cooking oil exhibited the presence of hydrocarbons and polymerized derivative of glyceride. It was observed that hydrocarbons such as n-hexadecane, 2,3,5,8-Tetramethyldecane, pentadecane, 2,6,10,14-tetra methylheptadecane, 3,3 dimethyl heptane, and 2,2,3,3,tera methyl pentane were detected in repeated cooking of oil. n-Teradecane might be formed by removal of terminal oxirane group from 2-Tetradecyl oxirane (Fig. 1). 2-Tetradecyl oxirane is highly unstable compound owing to presence of oxirane group which further dissociates from parent chain yield derivative of multiple hydrocarbons. The entire mechanism for generation of cyclic and noncyclic hydrocarbon in vegetable oil during repeated cooking is not possible to predict owing to spectrum of reactions and formation of unstable intermediates. However, major transformations proceed via a free radical mechanism of chain reactions. The hydrogen with the weakest bond on the carbon of oil was removed first and forms free radical, because low energy is required to break weak C–H bond. The free radical formations ensue more rapidly when weak C–H bond exists between two double bonds. This was due to withdrawing of electrons from C–H bond. Further, thermal reactions propagated in presence of atmospheric oxygen and form derivative of peroxides. The proposed mechanism is illustrated in Fig. 2. Lipid hydroperoxides had been identified as primary products of autoxidation, while decomposition of hydroperoxides, yields secondary oxidation products such as aldehydes, ketones, alcohols, hydrocarbons, volatile organic acids, and epoxy compounds (Shahidi and Zhong 2005). Susheelamma et al. (2002) reported a constant increase of peroxide value during three successive frying of all investigated samples of oils and blends. Simultaneously, water acts as weak nucleophile for ester linkage, whereas heat mass transfer and induced oxygen proliferate thermal oxidation. Incorporation of activated oxygen to double bond position triggers a series of complex reactions which might results in the formation of 2,3,-dimethyl-3-undecanol, oxiraneundecanoic acid and Triacontanoic acid methyl ester. (Fig. 3). Kamal-Eldin et al. (2003) described a similar mechanism of unsaturated fatty acids and lipid molecules via autoxidation and thermal oxidation, whereas Chung et al. (2004) used headspace oxygen method to evaluate oxidative stability of fats and oils. Genot et al. (1994) monitored the role of oxygen in lipid modification through measuring the rate of oxygen consumption using microcathode oxygen electrodes coupled to a computerized data collection and processing unit.

Zhang et al. (2009) reported Amadori and Diels–Alder rearrangements that lead to formation of advanced glycation end-products, glycation of protein and sugar-derived protein adducts during repeated cooking. The appearance of acrylamide, hydroxymethylfurfural and hydrocarbonacrylamide in course of cooking due to the condensation of reducing sugar and amino group causes potential health risk showing symptoms of cardiovascular, renal, and neurodegenerative disease. Thereby, many reputed restaurants and health concern food industries recommend vegetable oil for single cooking rather than repeated cooking.

### Identification of lipase hydrolyzed products

The extracellular enzyme produced from *P. chrysogenum* was very specific to hydrolysis of waste cooking oil because of the substrate used in fermentation. Firstly, the substrate used to obtain extracellular lipase was grease waste. Grease is semi-solid adhesive substrate did not allow the bacteria to that grow on it whereas *P. chrysogenum* had mycelium flourished well after seven days of fermentation (Supplementary). Secondly, the component of grease waste consist of derivatives of hydrocarbons and fatty acids. Similarly, cooking oil waste also consists of hydrocarbons and ester bonds of fatty acids. Therefore, lipase from *P. chrysogenum* was more specific for the hydrolysis of waste cooking oil. Adamczak and Bednarski (2004) had reported hydrolytic ratio of lipase that catalyzes beef tallow as 73 and 65 % from *Rhizomucor miehei* and *Yarrowia lipolytica,* respectively.

**Fig. 4:**
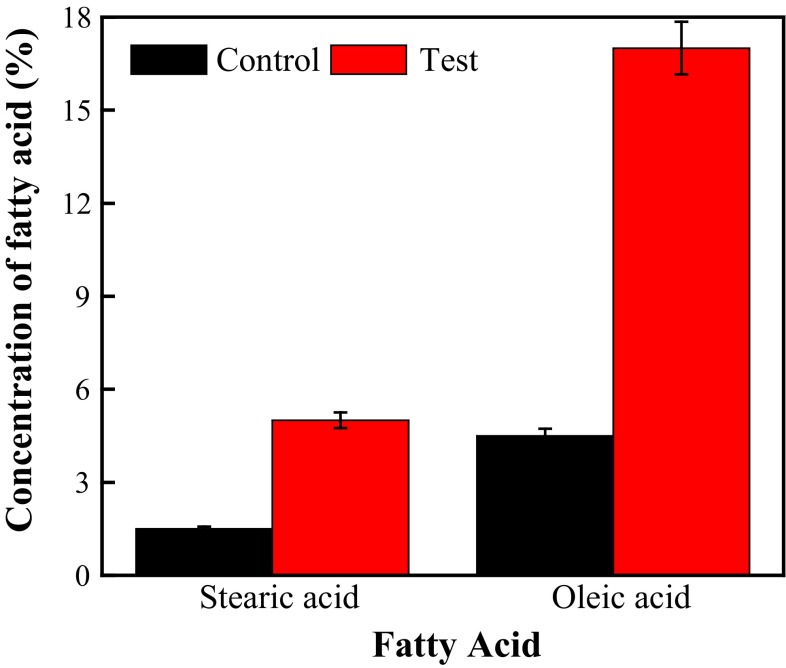
Identification of fatty acids by gas chromatography

The appearance of long chain fatty acids, oleic and stearic acids, was detected by gas chromatograph. It was observed that waste cooking oil treated with lipase yielded 17 % oleic acid and 5 % stearic acid (Fig. 4). The total 22 % C-18 fatty acid released from waste cooking oil indicated lipolytic efficiency of lipase. Initially, the concentration of linoleic acid in uncooked oil was 55–60 % and reduced to 28–32 % after repeated cooking of oil (Table 1). Approximately 30 % loss of linoleic acid indicates rapid oxidation to either oleic acid or stearic acid through proposed mechanism (Fig. 2). Thus, polyunsaturated linoleic acid containing double bond at carbon position 9 and 12 was to be reduced either to monounsaturated oleic acids or saturated stearic acid through oxidative free radical mechanism. However, these fatty acid were not detected during GC-MS analysis of waste cooking oil, which means multiple oxido-reduction process facilitated the formation of its conjugated derivates. Therefore, percentage of fatty acid in repeated heating of oil was decreased but the percentage of fatty acid in lipase-treated waste cooking oil was increased compared to untreated waste cooking oil. This might be due to hydrolysis of polymerized derivatives of fatty acid by lipase. Kowalski (2007) reported concentration of total fatty acids was decreased from 86 to 75 % after 72 h and to 68 % after 120 h heating of olive oil at 90 °C, while sunflower oil consisting initially 80 % fatty acid reduced to 61 % after heating for 72 h and to 56 % after 120 h. The lower loss of total fatty acids from olive oil compared to sunflower oil was because of its lower content of linoleic acid, which is readily oxidized. Edwinoliver et al. (2010) had used lipase to hydrolyze tallow and observed 36 % oleic acid, 19 % stearic acid and 27 % palmitic acid through gas chromatography.

**Table 1:** C-18 fatty acids composition in uncooked and repeated cooking oil

S. no.	Fatty acids	Fatty acid (%)	
		Before use	After repeated cooking
2	Oleic acid	4–6	4–5
3	Stearic acid	3–5	1–2
4	Linoleic acid	55–60	28–32

## Conclusions

Repeatedly used cooking oil consisted of various polymerized derivatives, hydrocarbons and glyceride molecules; its consumption is susceptible to health concern. It cannot be disposed due to environment issue. Therefore, lipase produced from *Penicillium chrysogenum* was exploited to hydrolyze waste cooking oil in order to procure value-added products as C-18 fatty acids. The 22 % C-18 fatty acid, which was obtained from waste cooking oil opened new avenues to explore lipase for various industrial applications such as for biofuels, soap and detergent industries. Current approach was very ecofriendly and profit-making under the principles of green chemistry.

## Electronic supplementary material

Below is the link to the electronic supplementary material.

Supplementary material 1 (DOCX 831 kb)
