# Supplementary figures and images for: Transformation of waste cooking oil into C-18 fatty acids using a novel lipase produced by Penicillium chrysogenum through solid state fermentation
Source: 3 Biotech. 2014 Dec 4;5(5):847–51. doi: 10.1007/s13205-014-0268-z (PMC4569631; doi:10.1007/s13205-014-0268-z)

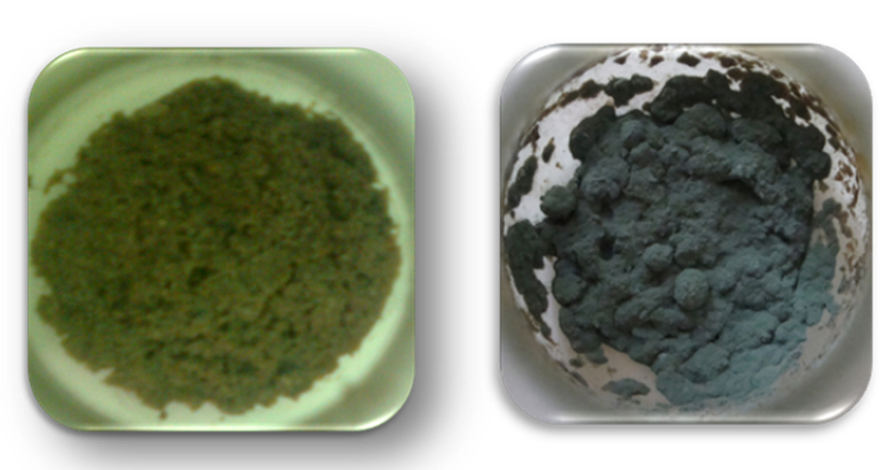


**Fig. 4 Fermenting media of lipase before and after fermentation**

Supplement: Supplementary file 1 — Supplementary material 1 (DOCX 831 kb) [file 13205_2014_268_MOESM1_ESM.docx]
